# Ecological Drivers of Biogeographic Patterns of Soil Archaeal Community

**DOI:** 10.1371/journal.pone.0063375

**Published:** 2013-05-22

**Authors:** Yuan-Ming Zheng, Peng Cao, Bojie Fu, Jane M. Hughes, Ji-Zheng He

**Affiliations:** 1 State Key Laboratory of Urban and Regional Ecology, Research Center for Eco-Environmental Sciences, Chinese Academy of Sciences, Beijing, China; 2 Environmental Futures Centre, Griffith School of Environment, Griffith University, Nathan, Queensland, Australia; Graz University of Technology (TU Graz), Austria

## Abstract

Knowledge about the biogeography of organisms has long been a focus in ecological research, including the mechanisms that generate and maintain diversity. In this study, we targeted a microbial group relatively underrepresented in the microbial biogeographic literature, the soil Archaea. We surveyed the archaeal abundance and community composition using real-time quantitative PCR and T-RFLP approaches for 105 soil samples from 2 habitat types to identify the archaeal distribution patterns and factors driving these patterns. Results showed that the soil archaeal community was affected by spatial and environmental variables, and 79% and 51% of the community variation was explained in the non-flooded soil (NS) and flooded soil (FS) habitat, respectively, showing its possible biogeographic distribution. The diversity patterns of soil Archaea across the landscape were influenced by a combination of stochastic and deterministic processes. The contribution from neutral processes was higher than that from deterministic processes associated with environmental variables. The variables pH, sample depth and longitude played key roles in determining the archaeal distribution in the NS habitat, while sampling depth, longitude and NH_4_^+^-N were most important in the FS habitat. Overall, there might be similar ecological drivers in the soil archaeal community as in macroorganism communities.

## Introduction

Biogeography is the study of the distribution of biodiversity over space and time [1]. For decades, knowledge about the biogeography of organisms has been a focus in ecological research, including the mechanisms that generate and maintain diversity, i.e., speciation, extinction, dispersal and species interactions [2]. It has been shown that macroorganisms have obvious zonal distributions along gradients of water and energy [3]. Whether or not microorganisms follow similar distribution patterns to macroorganisms is still a matter of debate [4], although we have benefited from recent advances in molecular biological techniques which make it possible to examine geographic distributions of microbes [1], [5], [6]. Some interesting studies on bacterial communities have been undertaken through comparative studies across different habitats [7]–[9]. While at a global range, archaea distribution were either mainly driven by salinity along a broad environmental gradient and habitat types [10], or precipitation gradient and vegetation cover [11]. However, there is insufficient information on soil Archaea from various locations and habitats, even though the diversity and composition of archaeal communities are thought to have a direct influence on a wide range of ecosystem processes [12]–[14], [15].

Moreover, a question, which prevails in the macroecology, is whether natural communities obey general predictable processes through species sorting in a spatially heterogeneous environment [16], [17], or communities are structured by neutral drift in species’ densities [18], [19]. This question mainly concerns the processes that structure ecological communities and the ecological mechanisms driving these patterns, defined as niche theory and neutral theory. The basic assumption of niche theory is that species differ in their traits to avoid competition and enable them to co-exist within communities for long periods of time [20]–[22]. This theory emphasizes the species-specific differences in explaining patterns in community organization and biodiversity. It predicts that species relative abundances will follow geometric series, the broken stick or some other niche-based models [23], [24]. In contrast, neutral theory emphasizes the equivalence of species in a community and the importance of stochastic events such as dispersal, local extinction and speciation [18], [19], [25]. No single species is at a competitive advantage or disadvantage, and exclusion does not occur [19], [26], [27]. Consequently, stochastic drift and changes in species composition will be only related to the geographic distances as a result of dispersal limitation [28]. Both theories have gained support from empirical studies [17], [29]–[34]. And thus, the synthesis of biogeographical theory with microbial ecology should be developed [35].

Here we address the question: what is the relative importance of stochastic and deterministic processes in structuring distribution patterns of Archaea in different environments? The biogeography of macroorganisms is much better studied and ecologists who study microorganisms and those who study macroorganisms have been interacting more often in recent years [1], [36]–[38], particularly in understanding mechanisms of community assembly. Nowadays, many efforts attempt to characterize the global microbial distribution patterns and the underlying driving mechanisms. For instance, the Earth Microbiome Project (EMP) is proposed to map the spatiotemporal variability of microbial across the globe and the hypothesis is that certain environmental features are correlated with specific combinations of microbial species [39]. However, the truth is the number of such studies available is limited because few microbial biogeographic studies report the geographic distance between their samples, or directly test for a distance effect relative to a contemporary environmental effect, or significant influences from longitude and latitude when test for the distance effect in their studies [1], [40]–[42]. Thus, the relative importance of stochastic and deterministic processes in structuring distribution patterns of Archaea in different environments is still unknown.

The legacy of historical separation, which means the dispersal limitation, will exert a dominant influence on the microbes compared with environmental factors as long as the research scale is big enough [1]. Also, ecologists often focus on the importance of temporal and spatial scales in their investigation. This leads to the second question: what is an appropriate spatial scale to explore the biogeography of microorganisms and its driving mechanisms? It is thought to be at the intermediate spatial scale (10–3000 km) that the influence of both historical contingencies and contemporary ecological factors on microbial biogeography is most likely to be detected [1], especially comparing the relative contribution of environmental or spatial variables [43].

China’s climates range from tropical to alpine, and various soil habitats have developed under different bioclimatic conditions within this vast area. This natural variety of different conditions may help to probe into the soil microbial biogeography. Our previous studies have investigated differences in soil bacterial diversity, which are mainly driven by historical contingencies, such as locations and soil depth [44]. In the present study, we collected 105 soil samples from two typical habitats, non-flooded soil (natural soil, NS) and flooded soil (paddy soil, FS), in China, which were separated at intermediate scales to clarify the biogeography of archaeal communities and examine the dominant ecological mechanism (niche or neutral theories) structuring microbial communities of Archaea. The FS habitat was attributed with quite different conditions from the ambient environment (air, water and soil). Our classification of two habitats might be helpful to test whether the FS habitat could be a “well-isolated habitat” [45], which meant the microorganisms inhabiting it were adapted to the conditions quite different from the ambient environment (the surrounding non-flooded soil) and geographical isolation might be one of the important components of microbial diversity, or at least compared the influences of two habitats on microorganisms. Molecular community profiling - terminal restriction fragment length polymorphism (T-RFLP) and quantitative real-time PCR was used to characterize the abundance, diversity, and distribution of archaeal communities to conduct a comparative study to distinguish the mechanisms in the individual habitats. Afterwards, the relationships between the soil archaeal communities and the spatial heterogeneity of the environmental factors were analyzed based on ecological models and multivariate statistical methods to tackle the mechanisms that generate the spatial patterns of microbial biodiversity.

## Methods

### Description of the Site and Sampling Design

**Figure 1:**
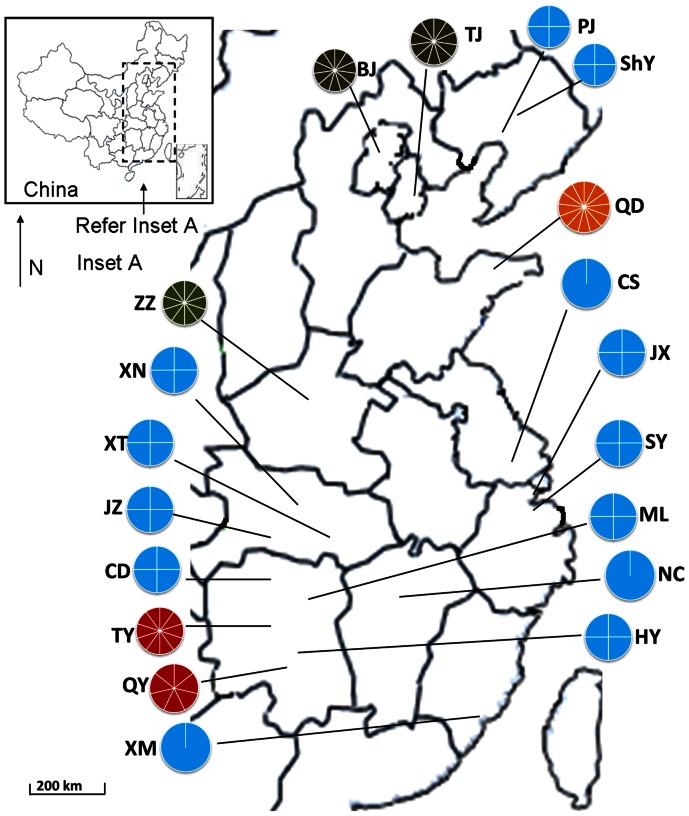
Soil sample locations as shown in a Chinese map. Different colors and numbers of sectors in the pie diagrams represent the soil type and the number of samples at each site. Coffer = cinnamon soil (ustic cambosols), orange = brown soil (udic agrosols), green = fluvo-aquic soil (aquic inceptisol), red = red soil (udic ferrosols), and blue = paddy soil.

In this study, two habitats were involved in the investigation, i.e., non-flooded soil (natural soil, NS) and flooded soil (FS) (In this study, no specific permissions were required for the locations/activities since the investigation fields did not belong to the protect areas and private lands, and the field studies did not involve endangered or protected species). There were 6 sites chosen for the NS and 13 sites for the FS habitat along a latitudinal gradient from the north to the south of China considering the longitudinal variation at the same time. Fifty-nine of the NS samples were from our previous work [46], and three new ones from BJ, TJ and QY were added. Thus totally sixty-two NS samples were obtained. The distance between any two sites ranged from 6 m to 1,873,226 m (Fig. 1). One hundred and five samples were obtained in total, and site information is listed in Table S1 (*Supplementary material*). Each soil sample was passed through a 2.0-mm sieve, and then stored at 4°C until analysis of soil characteristics. A subsample was taken from each sample and stored at −80°C for DNA extraction.

### Soil Chemical Analysis

Soil pH was determined with a soil to water ratio of 1: 2.5. Soil organic carbon (SOC) was determined using the K_2_Cr_2_O_7_ oxidation method [47]. Total N (TN) was determined using the Dumas method with an Element Analyzer (Vario EL III, Elementar, Hanau, Germany) [48]. Soil nitrate (NO_3_^–^N) and ammonium (NH_4_^+^-N) were extracted with 2 M KCl and determined with a Continuous Flow Analyzer (SAN++, Skalar, Breda, Holand). All results are listed in Table S2 (*Supplementary material*).

### Archaeal Abundance and Community Analysis

Soil DNA was extracted using MoBio UltraClean™ soil DNA isolation kits (MO BIO Laboratories, Inc., San Diego, CA, USA) according to the manufacturer’s protocol. Real-time PCR was performed on an iCycler iQ 5 thermocycler (Bio-Rad Laboratories, Inc., Hercules, CA, USA). The total number of archaeal 16S rRNA gene copies was determined according to the protocol of Cao et al [46]. The amplification efficiency for all qPCR reactions ranged from 91.7% to 94.9%. The specificity of amplification products was verified by melting curve analysis and standard agarose gel eletrophoresis. Standard curves for the qPCR assays were generated as described previously, using primer pairs Ar4F [49]/Ar958R [12] to amplify the 16S rRNA gene from soil DNA [46]. The PCR products were cloned into the pGEM-T Easy Vector (Promega, Madison, WI, USA). Plasmids from the positive clones with the targeted gene insert were extracted for sequencing and used as standards for the calibration curve. The plasmid concentration was 51.21 ng·µL^−1^, determined on a Nanodrop® ND-1000 UV-Vis Spectrophotometer (NanoDrop Technologies, Wilmington, DE, USA). Then, the plasmids were ten-fold serial diluted and used as templates with a final content of 1.02×10^−7^ to 1.02 ng in 25-µl reaction mixtures.

Archaeal community analysis used terminal restriction fragment length polymorphism (T-RFLP), and the process was described previously [46]. The PCR amplification for T-RFLP analysis was carried out using the archaeal primer pairs A364aF/A934bR [50] with the 5′ end of the A934bR primer labeled with 6-carboxyfluorescein (FAM). The purified FAM-labeled PCR product was digested by Hha I (TaKaRa Bio, Otsu, Shiga, Japan). The mixtures of the purified products and the internal standard GeneScan-1000 ROX (Applied Biosystems, Foster City, CA, USA) were denatured for 3 min at 95°C, and the DNA fragments were size separated using a 3130*xl* Genetic Analyzer (Applied Biosystems, Foster City, CA, USA). T-RFLP profiles were produced using the GeneMapper software (version 3.7; ABI, USA), and peaks at positions between 50 to 550 bp were selected because most T-RFs fall in this range and also to avoid T-RFs caused by primer-dimers. The relative abundance of a T-RF was calculated by dividing the peak height of the T-RF by the total peak height of all T-RFs in the profile. In addition, we also calculated the relative abundance of a T-RF using peak area, and there was no significant difference comparing with the results of peak height. The peaks with height ≤1% of the total peak height were not included in further analyses.

### Data Analysis

Prior to analysis, GPS coordinates were converted to UTM coordinates in meters for principal coordinates of neighbor matrices (PCNM) and Mantel test (vegan library in R software, 2010). These spatial data and other environmental variables (Table S2) were standardized (mean = 0, standard deviation = 1) for the redundancy analysis (RDA) with manual forward selection (CANOCO 4.5, CANOCO 4.5, Centre for Biometry Waginingen, The Netherlands). Log normalized T-RFLP profiles data were subjected to the PCNM with variation partitioning, Mantel test and RDA analyses. PCNM with variation partitioning and Mantel tests based on distance dissimilarity matrices. RDA analyses directly used log normalized T-RFLP profiles data.

The results of real-time PCR were firstly converted into cell numbers based on the average number of 16S rRNA gene copies for Archaea (1.77) [51]–[52]. The number of distinct T-RFs was used as an estimate of species richness, so the proportion of T-RFs within a sample combined with real-time PCR results represented the abundance of T-RFs species; this is not a taxonomic definition of the true number of species within a sample, and the term T-RF species was applied in recognition of that fact. The most widely used α, β-diversity indices, i.e., Shannon-Wiener index, Simpson index, Evenness index and Bray-Curtis dissimilarity index, were calculated in the R statistical language. For a diversity measure, the mean value per site (e.g., n = 10 for BJ) was used for further analysis. For the β-diversity measure, the mean pair-wise measure between samples for each site was used. Sites with only single sample were omitted from all analysis of β-diversity. The Welch test was used to compare archaeal abundance, T-RFs species richness, Shannon-Wiener index, Simpson index, Evenness index and Bray-Curtis dissimilarity index between the two habitats, NS and FS (SPSS 13.0, IBM Co., Armonk, New York, USA). *P*<0.05 was considered to be significant.

In order to test the sampling efficiency, T-RFs accumulation curves were computed using specaccum in R (vegan library in R software, 2010) [24]. Three kinds of methods were used to analyze the effects of environmental variables and spatial structure on archaeal community variation: variance partitioning using extracted PCNM as spatial predictors employing RDA, correlation of archaeal ß-diversity and the change of spatial and environmental variables using the Mantel test, and manual forward selection RDA to explain the proportion of the variance in archaeal community explained by each significant variable. For the Mantel test, the dissimilarity index of spatial and environmental variables between each pair of sites was calculated using Euclidean distances when tested by Mantel analysis and the importance of each variable was evaluated using spearman correlation coefficient. PCNM with variation partitioning analysis partitioned the variation represented by adjusted R^2^ values (Ra^2^) into four fractions as Dumbrell described: (a) variation explained by environmental variables and not spatially structured, (b) variation explained by environmental variables with spatial structure, (c) spatially structured variation not explained by the environmental variables and (d) residual variation [45]. Thus 12 of the 34 extracted PCNM variables that significantly (α = 0.05) explained the spatial structure of archaeal community in NS, and 8 of 24 PCNM variables could be used to explain the spatial structure in FS (all the PCNM variables were examined by forward selection based on 10 000 permutation test). The PCNM analysis used ‘PCNM’ and ‘vegan’ libraries in the R statistical language. Mantel analysis was based on 10 000 randomizations of the original data. Finally, the relationship between archaeal community and both environmental and spatial variables was analyzed using RDA with manual forward selection using 10 000 Monte Carlo permutation tests.

## Results

### Archaeal Abundance and Alpha, Beta Diversity

The soil pH, SOC and TN were significantly different among the sampling sites. Soil pH varied widely in NS with the following order: ZZ> TJ> BJ> QD> QY> TY (Table S2). The pH also varied between 5.63 and 9.08 among different sites in FS (Table S2). Soil samples of PJ had the highest pH (9.08±0.14) while TY soils had the lowest pH (4.42±0.34). SOC and TN varied with different site and ranged from 4.19 to 62.2 and 0.33 to 8.87, respectively (Table S2). The archaeal abundance ranged from 8.72×10^6^ to 4.12×10^7^ cells g^−1^ dry soil in NS, which was significantly higher than in FS which ranged from 3.72×10^5^ to 1.60×10^6^ cells g^−1^ dry soil (*P* = 0.01) (Table 1). In total, 12 and 19 T-RFs were detected in NS and FS, respectively. In NS, the dominated T-RFs were 162 bp, 192 bp, 231 bp and 537 bp with the relative abundance ranging from 0.00% to 35.5%, 11.3% to 88.7%, 0.00% to 71.1% and 0.00% to 62.8%, respectively. The dominated T-RFs in FS were 81 bp, 86 bp, 117 bp, 162 bp, 192 bp, 207 bp, 227 bp and 539 bp varied between 1.09–25.6%, 1.32–43.2%, 1.41–10.5%, 1.10–21.9%, 9.84–86.2%, 1.06–14.1%, 1.18–42.0% and 1.81–63.1%, respectively. As to the archaeal community biodiversity, T-RFs species richness (*P* = 0.00), Shannon-Wiener index (*P* = 0.00) and Simpson’s index (*P* = 0.01) were significantly lower in NS than in FS, but there were no significant differences for evenness (*P* = 0.32) or β-dissimilarity indices (*P* = 0.19) between the two habitats (Table 1). For the NS habitat, the maxima of the richness, Shannon-Wiener and Simpson indices were found in soil at TY, while the minima were found at TJ. For the FS habitat, the maxima of the richness, Shannon-Wiener and Simpson indices were attained at NC, while the minimum values at ShY (Table 1).

**Table 1:** Abundance and diversity of archaeal communities in different sampling sites.

Sample name	Abundance*(cells g^−1^ soil)	α- diversity indices				β- dissimilarity indices
		Richness*	Shannon–Wiener*	Simpson*	Evenness	
NS						
BJ	2.34E+07	3.73	0.94	0.53	0.71	0.66
TJ	4.12E+07	3.64	0.90	0.52	0.68	0.48
QD	1.15E+07	4.46	1.01	0.54	0.69	0.44
ZZ	3.67E+07	5.10	1.13	0.56	0.70	0.38
TY	1.57E+07	4.50	1.17	0.63	0.78	0.54
QY	8.72E+06	4.43	1.01	0.54	0.69	0.68
FS						
PJ	4.55E+05	8.75	1.48	0.64	0.68	0.49
ShY	3.72E+05	6.00	0.95	0.46	0.54	0.32
CS	1.60E+06	9.00	1.34	0.59	0.61	0.00
XT	9.14E+05	9.00	1.13	0.51	0.57	0.17
JZ	8.23E+05	7.75	1.34	0.62	0.66	0.65
XN	4.13E+05	7.50	1.40	0.65	0.71	0.65
JX	6.62E+05	8.00	1.44	0.67	0.69	0.50
SY	5.31E+05	9.25	1.66	0.74	0.76	0.53
CD	6.94E+05	7.25	1.47	0.69	0.74	0.85
ML	1.53E+06	7.75	1.30	0.64	0.64	0.42
HY	1.21E+06	6.25	1.39	0.68	0.76	0.67
NC	7.56E+05	9.00	1.67	0.77	0.76	0.00
XM	3.82E+05	8.00	1.56	0.76	0.75	0.00

* Values differ at *P*<0.05 between NS and FS habitat using the Welch test.

Values are mean for each site.

NS, non-flooded soil (natural soil); FS, flooded soil.

### The Influence of Spatial and Environmental Effects on Archaeal Communities

**Figure 2:**
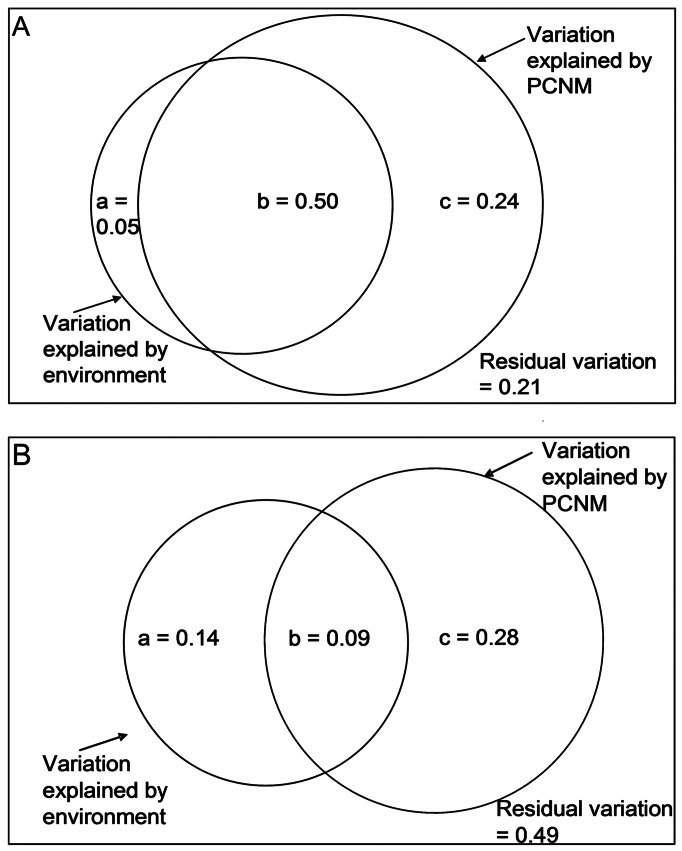
Variation partitioning results. Venn diagrams represent the partitioning variation in the archaeal community (beta- diversity) in different habitats (A) non-flooded soil (NS) and (B) flooded soil (FS). Each box represented 100% of the variation in the corresponding response variable, and the reported fractions were adjusted R^2^ statstics (Ra^2^).

T-RFs species accumulation curves showed that they had asymptotic trends for the data from both NS and FS (*Supplementary material* Fig. S1). Therefore, further sampling would be unlikely to qualitatively affect the results. PCNM with variation partitioning analysis has been shown to be a successful method to examine the relative contribution of spatial structure and environmental variables to the variation of ecological communities [54]–[57]. There was 79% of the archaeal community variation explained in the NS habitat using adjusted R^2^ values (Ra^2^) (a+b+c; F = 2.11; *P*<0.001) (Fig. 2A). 5% of the explained variation in community composition was attributed to environmental variables (a) and 24% to spatially structured variation (c). The majority of variation explained was spatially structured environmental variables (b, 50%). In FS, analysis of variation partitioning explained 51% of the variation of archaeal communities (a+b+c; F = 2.05; *P*<0.001) (Fig. 2B). The explained variation in community composition was attributed to environmental variables (a+b) and spatially structured variation (b+c), which was 23% and 37%, respectively. This indicated that the majority of variation in archaeal community composition was explained by spatial structure in the FS habitat.

Mantel tests showed that environmental variables were positively correlated with β-diversity measured by Bray-Curits’s dissimilarity index (r = 0.22, *P* = 0.002). β-diversity of NS was positively correlated with pH, then profile depth (cm), longitude (m), NH_4_^+^-N, and altitude (m) (Table 2). However, in the FS habitat, β-diversity was significantly correlated only with depth (Table 2). Combined with the results of PCNM analysis, β-diversity of the archaeal community was predominantly controlled by spatial structure both in NS and FS habitats.

**Table 2:** Relationship between environmental/geographic variables and beta diversity of archaeal communities using Mantel test and archaeal community composition using redundancy analysis (RDA).

	Non-flooded soil		Flooded soil	
	Mantel test	RDA	Mantel test	RDA
	Bray-Curits’s index	Manual forward selection	Bray-Curits’s index	Manual forward selection
	R value	F value	R value	F value
pH	0.32**	23.20**	ns	ns
SOC	ns	ns	ns	ns
TN	ns	ns	ns	ns
NO_3_^–^N	ns	ns	ns	ns
NH_4_^+^-N	0.16**	ns	ns	4.47*
Latitude	ns	ns	ns	ns
Longitude	0.20**	6.70**	ns	6.68**
Altitude	0.11*	ns	ns	ns
Depth	0.20**	14.75**	0.26**	10.06**

* Values differ at *P*<0.05;

** Values differ at *P*<0.01.

ns, not significant.

**Figure 3:**
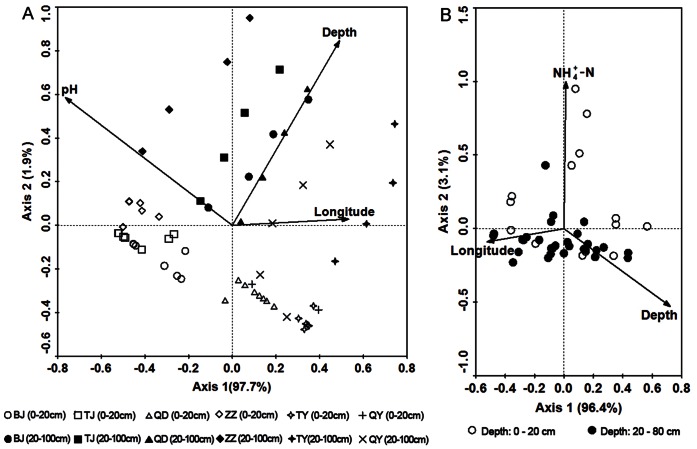
Redundancy analysis (RDA) for the relationship between environmental or geographic variables and archaeal community composition. (A) non-flooded soil (NS) and (B) flooded soil (FS).

In NS and FS habitats, RDA significantly explained 98.1% and 97.3% respectively of the species-environment relationship across the first two canonical axes under the full model that included all environmental variables. With manual forward selection of environmental variables using Monte Carlo permutation tests, RDA of NS revealed that soil pH, depth and longitude were significantly related to the archaeal community composition (Table 2), which accounted for 28%, 14% and 6% of the variation in community composition, respectively. Latitude was excluded, and the axis 1 explained 97.7% of the species-environment relationship, which was much higher than our previous results [46]. Depth, longitude and NH_4_^+^-N were significant variables in the FS habitat (Table 2), accounting for 20%, 11% and 7% of the variation in community composition, respectively. It showed that these variables explained 99.6% and 99.5% of the variation within the species–environment relationship across the first two canonical axes in different soil habitat (Table 3; Fig. 3). No other environmental variable was significantly related to archaeal community composition (*P*>0.05 in all the cases).

**Table 3:** Summary results of the redundancy analysis (RDA) for archaeal community composition.

	Fig. 3A			Fig. 3B		
		Axs1	Axs2		Axs1	Axs2
Eigenvalue		0.472	0.009		0.369	0.012
Species-environment correlations		0.742	0.440		0.661	0.485
Cumulative percentage variance of species data		47.2%	0.9%		36.9%	1.2%
Cumulative percentage variance of species-environment relation		97.7%	1.9%		96.4%	3.1%
Weighted correlation	pH	−0.568	0.258	Depth	0.478	−0.257
	Longitude	0.395	0.013	Longitude	−0.345	−0.044
	Depth	0.364	0.372	NH_4_^+^-N	0.008	0.485

## Discussion

In our study, real-time PCR and T-RFLP were used to analyze the abundance and diversity of archaeal communities. We found large differences between the NS and the FS habitats using abundance and α-diversity indices as indicators. These differences were similar to the comparative study on soils from forest and floodplain by Kemnitz et al. [50], [58]. Both the Shannon-Wiener index and the Simpson index are influenced by the richness and the evenness of T-RFs species in the sample. Larger values of each of these indices indicate the higher diversity [24]. Thus the FS habitat was more diverse than the NS habitat, which might indicate that FS would be more stable when experiencing disturbance due to its more complex structure [24]. However, there was no significant difference in β-diversity of archaeal communities between the NS and FS habitats based on the results of this study. This was mainly because of the big differences within each habitat, i.e., the differences between the two groups was as great as the differences within each group. These results also suggested that archaeal communities varied with location or site. β-diversity needs to be explored using more complex multivariate statistical methods, as suggested by Legendre et al. [59].

In the NS habitat, soil pH, depth of sampling and longitude were found to be responsible for the regulation of archaeal community, with soil pH the most important factor like our previous results [46], suggesting strong selective pressures. In contrast, longitude, sampling depth and concentration of NH_4_^+^-N were the most important variables in the FS habitat, with longitude the most important. Longitude, reflecting geographic distance, played a dominant role in both NS and FS habitats, which indicated the effects from dispersal limitation. Furthermore, in NS and FS habitats, explained variances accounted for 79% and 51% of total variation, respectively (a+b+c, Fig. 2). These explained proportions were mainly attributed to the stochastic processes, while the deterministic processes were relatively weak. The independent contribution of spatial variables was 24% and 28%, while the contribution of environmental variables was only 5% and 14% for the NS and FS habitat, respectively. Therefore, at the scale examined here, stochastic processes seem to be more important in structuring the community composition of Archaea, indicating some similarities with phytoecology [60]. To some extent it could be concluded that soil archaea community is controlled by spatial factors to certain extent, although only longitude was significant in our study.

It was notable that the portion of variation undetermined in FS was 49% (fraction d in Fig. 2). Although the underlying processes could not be identified from the available data, analyses implied that they could be (at least partly) independent of the measured environmental variables (which obviously were not exhaustive, and did not include all of the possible environmental variables in nature), and a fair amount of variation was due to local effects of unmeasured (biotic or abiotic) controlling variables, or to spatial structures that have been missed because they required more complex functions to be described [61]. Another interpretation is that it might be due to stochastic processes. The latter explanation has theoretical connection to the neutral theory of macroecology that assumes the dynamics of populations are primarily driven by ecological drift and dispersal with or without limitation, and are not habitat dependent. Dispersal has a spatial signature and produces variation in fractions (c) and (d) whereas the effect of drift comes out in fraction (d) [60]. Indeed, the contemporary factors chosen might be arbitrary sometimes. This would impact the statistical results significantly. For example, there was no relationship between pH and the archaeal community in FS, which contrasts not only with NS, but also with other studies [6], [8]. For the FS habitat, some other variables might be considered, such as reduction potential (*Eh*), to improve the explanation of variation in community composition, whereas for the NS habitat, the current set of variables produced satisfying results.

There was a clear difference between the two habitat types in the proportion of the variation accounted for by the combined effects of environmental and spatial variables. This proportion was as high as 50% in NS showing the environmental variables depended on the spatial structure, while in FS, there was no such obvious combined effect. Since in PCNM analysis, fraction (c) was related with the pure effects of neutral processes and fraction (a) represented the pure effects of niche differentiation, fraction (b) should indicate the interaction of niche and neutral theories in driving the soil archaeal biogeography. This is probably because niche and neutral processes are not diametrically opposed to each other and a community is likely determined by the interplay of the two processes, as ecologists in macroecology are acknowledging [7], [9], [54], [62]. Thus, different studies showed high variations in the driving patterns of organisms [34], even between microorganisms. Microbial ecological theory needs more exploration to distinguish the contribution of local niche-based processes and dispersal limitation.

As has been known for some time, factors driving macroorganism distributions are water-energy related. Biogeographic patterns of plants are dependent on longitude, latitude and/or altitude. In our study, because of wide variation in water-energy in the NS habitat, niche differentiation was significant [44], [62]. It has been suggested that species tend to differ in their traits in order to avoid competition and enable them to co-exist within communities for long periods of time [20]–[22]. In contrast, flooded conditions could make the FS habitats located in the different sites more homogeneous. Microorganisms in paddy fields might be less affected by environmental factors, especially considering that sampling was in summer. Therefore, weak niche differentiation possibly resulted in stronger effects of spatial structure. Conversely, the flooded environment may have made the FS habitats more isolated from one another as Papke et al. [45] described, which would have reinforced the effects of dispersal limitation. The fast metabolic rate and short generation time of microorganisms might also result in stochastic processes being more important in determining community assemblages [19]. This phenomenon caused by the unique land use pattern of paddy fields is potentially very useful and worthy of exploring further in the future.

In addition, as many environmental factors as possible should be included, especially in the preliminary investigation. Factors involved with the ecological drivers are complicated. Sometimes we could hardly distinguish the original variables from derived variables. Many studies pointed out that pH was a significant factor in driving the microbial distribution patterns [46], [53], [63]. However, it is still controlled by other soil properties, such as soil minerals. So whether this means that we should substitute pH with more original variables such as soil mineral composition? The same question will be arisen in the spatial variables selection. Whether we should only use longitude and latitude, or we should add moisture and temperature, which are considered the representative factor related to longitude and latitude, respectively? If moisture and temperature should be involved in the calculation of ecological driving processes, they should be regarded as spatial variables or environmental variables? So in future studies, we should pay more attention to the systematic classification of variables which are related to the effect analysis of spatial and environmental variables contribution to the community organization.

In conclusion, we applied ecological theories for macroorganisms to explore microorganism communities. Uniquely for a microbial community by a case study of archaeal communities across China, we partitioned the relative importance of deterministic and stochastic processes, and explained the patterns by niche or neutral theory. We suggest that the biogeographic patterns of soil Archaea and ecological mechanisms driving distributions were explained by the niche and neutral theories jointly in different terrestrial ecosystems, as well as found in macro ecological studies.

## Supporting Information

Figure S1 **T-RFs species accumulation curves.** T-RFs species accumulation is shown as archaeal T-RFs species abundance data sampled per non-flooded soil (NS) (A) or per flooded soil (FS) (B). Data points mean estimated T-RFs species richness (± SE) using rarefaction in the R statistical language.
(TIF)

Table S1 **Descriptive information of the soil samples.**
(DOC)

Table S2 **Basic chemical properties of the examined soils.**
(DOC)
