# Correction: Ecological Drivers of Biogeographic Patterns of Soil Archaeal Community

**DOI:** 10.1371/annotation/69333ae7-757a-4651-831c-f28c5eb02120

**Published:** 2013-10-29

**Authors:** Yuan-Ming Zheng, Peng Cao, Bojie Fu, Jane M. Hughes, Ji-Zheng He

These authors contributed equally to this work:

Yuan-Ming Zheng, State Key Laboratory of Urban and Regional Ecology, Research Center for Eco-Environmental Sciences, Chinese Academy of Sciences, Beijing, China

Peng Cao, State Key Laboratory of Urban and Regional Ecology, Research Center for Eco-Environmental Sciences, Chinese Academy of Sciences, Beijing, China 

