# Supplementary figures and images for: Ecological Drivers of Biogeographic Patterns of Soil Archaeal Community
Source: PLoS One. 2013 May 22;8(5):e63375. doi: 10.1371/journal.pone.0063375 (PMC3661566; doi:10.1371/journal.pone.0063375)

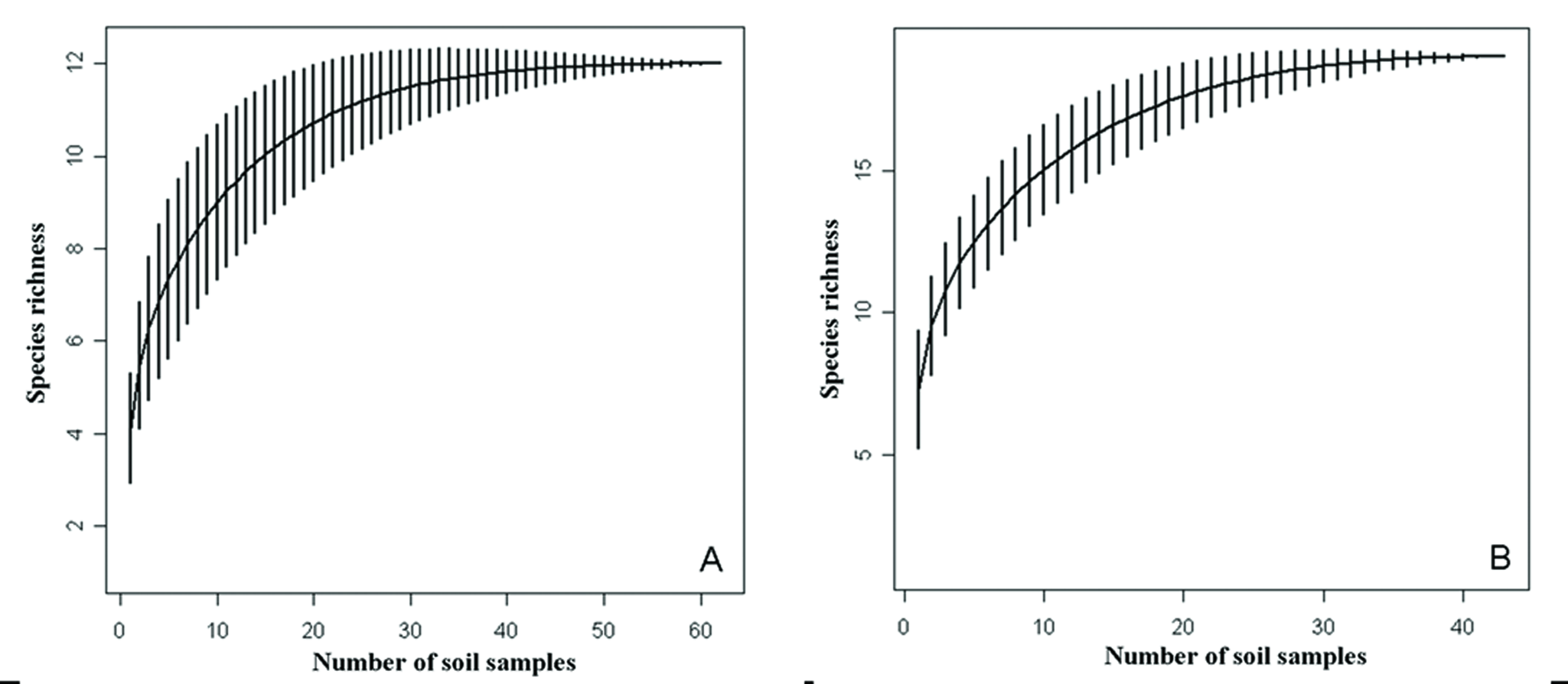

Supplement: Figure S1 — T-RFs species accumulation curves. T-RFs species accumulation is shown as archaeal T-RFs species abundance data sampled per non-flooded soil (NS) (A) or per flooded soil (FS) (B). Data points mean estimated T-RFs species richness (± SE) using rarefaction in the R statistical language. (TIF) [file pone.0063375.s001.tif]
