# Supplementary material for: Ecological Drivers of Biogeographic Patterns of Soil Archaeal Community
Source: PLoS One. 2013 May 22;8(5):e63375. doi: 10.1371/journal.pone.0063375 (PMC3661566; doi:10.1371/journal.pone.0063375)
Supplement: Table S1 — Descriptive information of the soil samples. (DOC) [file pone.0063375.s002.doc]

Table S1. Descriptive information of the soil samples ^a^

| Sites | ^b^Depth (cm) | N | Location | Altitude (m) | Landuse | Sampling time |
| --- | --- | --- | --- | --- | --- | --- |
| BJ (Beijing) | 0-20 | 5 | 116º48´-116º54´E  40º25´-40º29´N | 72.0-175.0 | Upland soils | May, 2009 |
|  | 0-100 | 6 |  |  |  |  |
| TJ (Tianjin) | 0-20 | 5 | 117º28´-117º31´E  40º04´-40º07´N | 35.0-218.0 | Upland soils | May, 2009 |
|  | 0-100 | 6 |  |  |  |  |
| QD (Qingdao, Shandong) | 0-20 | 7 | 120º31´-120º37´E  36º17´-36º18´N | 29.0-78.0 | Upland soils | June, 2009 |
|  | 0-100 | 6 |  |  |  |  |
| ZZ (Zhengzhou, Henan) | 0-20 | 4 | 113º39´E  34º43´-34º53´N | 84.0-103.0 | Upland soils | June, 2009 |
|  | 0-100 | 6 |  |  |  |  |
| TY (Taoyuan, Hunan) | 0-20 | 4 | 111º26´E  28º55´N | 104.0-108.0 | Upland soils | June, 2009 |
|  | 0-100 | 6 |  |  |  |  |
| QY (Qiyang, Hunan) | 0-20 | 1 | 111º52´E  26º45´N | 151.0-162.0 | Upland soils | June, 2009 |
|  | 0-100 | 6 |  |  |  |  |
| PJ (Panjin Liaoning) | 0-80 | 4 | 122°00´E  41°13´N | 7.0-8.0 | Paddy soils | August, 2010 |
| ShY (Shenyang, Liaoning) | 0-80 | 4 | 123°24´E  41°31´N | 42.0-43.0 | Paddy soils | July, 2010 |
| CS (Changshu, Jiangsu) | 0-20 | 1 | 120°42′E  31°33N | 3.0 | Paddy soils | July, 2010 |
| XT (Xiantao, Hubei) | 0-80 | 4 | 113º00´E  30º26´N | 29.0-30.0 | Paddy soils | July, 2010 |
| JZ (Jingzhou, Hubei) | 0-80 | 4 | 113º03´E  30º21´N | 26.0-27.0 | Paddy soils | July, 2010 |
| XN (Xianning, Hubei) | 0-80 | 4 | 114º21´E  30º10´N | 25.0-26.0 | Paddy soils | July, 2010 |
| JX (Jiaxing, Zhejiang) | 0-80 | 4 | 120º42´E  30º49´N | 5.0-6.0 | Paddy soils | July, 2010 |
| SY (Shangyu, Zhejiang) | 0-80 | 4 | 120º55´E  29º56´N | 6.0-7.0 | Paddy soils | July, 2010 |
| CD (Changde, Hunan) | 0-80 | 4 | 111º37´E  29º10´N | 48.0-49.0 | Paddy soils | July, 2010 |
| ML (Miluo, Hunan) | 0-80 | 4 | 113º2´E  28º44´N | 55.0-56.0 | Paddy soils | July, 2010 |
| HY (Hengyang, Hunan) | 0-80 | 4 | 112º53´E  27º10´N | 48.0-49.0 | Paddy soils | July, 2010 |
| NC (Nanchang, Jiangxi) | 0-20 | 1 | 115º22´E  28º46´N | 37.0 | Paddy soils | July, 2010 |
| XM (Xiamen, Fujian) | 0-20 | 1 | 117º58´E  24º37´N | 36.0 | Paddy soils | July, 2010 |

^a^ Refer to Cao et al. [46]. Three new samples were added from BJ, TJ and QY, respectively.

^b^ Depth, 0-20 cm means topsoils; 0-100 cm means the soil profiles with 6 layers, i.e. 0-10 cm, 10-20 cm, 20-40 cm, 40- 60 cm, 60- 80 cm, and 80-100 cm; 0-80 cm means the soil profiles with 4 layers, i.e. 0-20 cm, 20-40 cm, 40- 60 cm, and 60- 80 cm.
