# Supplementary material for: Ecological Drivers of Biogeographic Patterns of Soil Archaeal Community
Source: PLoS One. 2013 May 22;8(5):e63375. doi: 10.1371/journal.pone.0063375 (PMC3661566; doi:10.1371/journal.pone.0063375)
Supplement: Table S2 — Basic chemical properties of the examined soils. (DOC) [file pone.0063375.s003.doc]

Table S2. Basic chemical properties of the examined soils ^a^

| Habitat | Sample  name | pH ^b^  (H_2_O) | SOC ^b^  (g kg^-1^) | TN ^b^  (g kg^-1^) | NO_3_^-^-N  (mg kg^-1^) | NH_4_^+^-N  (mg kg^-1^) |
| --- | --- | --- | --- | --- | --- | --- |
| NS | BJ | 6.40±0.42 | 12.2±10.5 | 0.80±0.50 | 2.83±0.83 | 20.5±4.0 |
|  | TJ | 7.05±0.15 | 15.9±12.8 | 1.14±0.78 | 3.28±1.18 | 17.3±1.1 |
|  | QD | 5.46±0.58 | 62.6±3.9 | 8.12±.021 | 7.57±1.12 | 12.3±3.0 |
|  | ZZ | 8.19±0.30 | 9.84±6.11 | 0.67±.034 | 3.18±1.01 | 14.9±7.6 |
|  | TY | 4.42±0.34 | 15.1±8.9 | 0.97±0.35 | 5.15±3.48 | 23.0±4.7 |
|  | QY | 4.73±0.30 | 8.63±6.20 | 0.72±0.22 | 3.23±0.37 | 25.7±3.3 |
| FS | PJ | 9.08±0.14 | 4.19±1.91 | 0.33±0.11 | 0.29±0.14 | 9.66±1.69 |
|  | ShY | 7.19±0.25 | 5.76±2.44 | 0.41±0.11 | 1.95±1.04 | 20.6±7.8 |
|  | CS | 8.01±0.06 | 20.3±0.6 | 1.54±0.01 | 0.55±0.00 | 10.8±3.1 |
|  | XT | 8.38±0.13 | 10.2±2.6 | 0.69±0.24 | 2.75±1.92 | 9.65±2.16 |
|  | JZ | 7.07±0.37 | 6.73±4.72 | 0.53±0.32 | 2.25±0.65 | 8.41±3.43 |
|  | XN | 6.84±0.68 | 6.32±4.46 | 0.53±0.32 | 1.72±0.57 | 14.5±3.6 |
|  | JX | 7.30±0.50 | 5.65±5.26 | 8.87±0.41 | 1.27±0.38 | 14.6±14.0 |
|  | SY | 6.68±1.12 | 10.0±7.6 | 0.81±0.64 | 4.07±0.69 | 15.4±11.4 |
|  | CD | 6.65±0.21 | 4.71±3.39 | 0.44±0.22 | 2.13±0.23 | 13.6±3.4 |
|  | ML | 6.41±1.14 | 9.37±6.01 | 0.72±0.51 | 3.98±0.59 | 14.0±6.5 |
|  | HY | 6.64±0.95 | 8.40±7.30 | 0.62±0.49 | 2.08±0.63 | 13.5±6.0 |
|  | NC | 5.63±0.60 | 15.4±4.9 | 1.19±0.03 | 1.95±1.02 | 46.5±0.0 |
|  | XM | 6.72±0.41 | 16.6±6.3 | 1.22±0.03 | 0.73±0.21 | 53.3±20.1 |

^a^ For samples in NS habitat, please refer to Cao et al. [46]. Three new samples were added from BJ, TJ and QY, respectively. All of the data were re-analyzed.

^b^ Values differ at P < 0.05 among the sampling sites.

SOC was the abbreviation of soil organic carbon.

TN was the abbreviation of total nitrogen.
